# Detergent-Based Decellularization Preserves Extracellular Matrix Ultrastructure in Ovine Soft Tissues

**DOI:** 10.3390/biomimetics11050301

**Published:** 2026-04-26

**Authors:** Ibrahim E. Helal, Mahmoud F. Ahmed, Ahmed M. Abdellatif, Mohamed A. Hashem, Hatim A. Al-Abbadi, Elsayed Metwally

**Affiliations:** 1 Department of Agriculture, Faculty of Environment Sciences, King Abdulaziz University, Jeddah 21589, Saudi Arabia; ihelal@kau.edu.sa; 2 Department of Surgery, Anesthesiology and Radiology, Faculty of Veterinary Medicine, Suez Canal University, 4.5 Ring Road, Ismailia 41522, Egypt; mohamed.hashem@vet.suez.edu.eg; 3 Department of Surgery and Theriogenology, Faculty of Veterinary Medicine, King Salman International University, Ras Sudr 46612, Egypt; 4 Department of Anatomy and Embryology, Faculty of Veterinary Medicine, Mansoura University, Mansoura 35516, Egypt; abdellatif_ma@mans.edu.eg; 5 University Hospital, Faculty of Medicine, King Abdulaziz University, Jeddah 80212, Saudi Arabia; hatimalabbadi@yahoo.com; 6 Department of Cytology and Histology, Faculty of Veterinary Medicine, Suez Canal University, 4.5 Ring Road, Ismailia 41522, Egypt

**Keywords:** decellularization, biomaterials, extracellular matrix, ovine, tissue engineering, scanning electron microscopy

## Abstract

Decellularized extracellular matrix (dECM) scaffolds derived from xenogeneic tissues represent promising biomaterials for tissue engineering. In this study, dECM scaffolds were developed and characterized from four ovine tissues—skin, tunica vaginalis, fascia lata, and pericardium—using a detergent-based decellularization protocol to evaluate decellularization efficiency and extracellular matrix (ECM) preservation. Decellularization was performed using a sequential detergent-based protocol with sodium dodecyl sulfate and Triton X-100. Decellularization efficacy and matrix preservation were evaluated through gross examination, histological analysis, scanning electron microscopy (SEM), and residual DNA quantification. Gross inspection revealed increased translucency and reduced pigmentation in decellularized tissues compared with native counterparts, indicating effective cellular removal while maintaining overall tissue architecture. Histological assessment confirmed the complete absence of nuclear and cytoplasmic material, alongside preservation of collagen-rich extracellular matrix organization. SEM analysis demonstrated well-maintained ultrastructural features, including aligned collagen fibers and porous ECM architecture, with complete removal of epithelial and stromal cellular elements. Quantitative analysis revealed approximately 94% reduction in residual DNA content across all decellularized tissues compared with native controls. This study demonstrated that the employed detergent-based protocol reliably produces structurally preserved, acellular scaffolds from multiple ovine tissues. The resulting biomaterials exhibit structural characteristics that support their potential use in tissue engineering applications, pending further functional validation.

## 1. Introduction

Tissue engineering and regenerative medicine aim to restore or replace damaged tissues through the use of biomimetic scaffolds capable of supporting cellular adhesion, migration, proliferation, and differentiation [1,2]. Central to these strategies is the extracellular matrix (ECM), a complex three-dimensional network that provides not only structural support but also essential biochemical and biomechanical cues regulating cellular behavior [3,4]. Preservation of the native ECM architecture is therefore a critical requirement for the development of functional scaffolds in regenerative applications.

Decellularized extracellular matrix (dECM) derived from native tissues has emerged as a promising scaffold material, as it retains tissue-specific microarchitecture, biomechanical integrity, and bioactive signaling molecules while minimizing immunogenicity through removal of cellular components [5,6]. Xenogeneic tissues, particularly those of ovine origin, have gained increasing attention due to their anatomical similarity to human tissues, favorable mechanical properties, and widespread availability [7,8].

Ovine soft tissues such as skin, tunica vaginalis, fascia lata, and pericardium are characterized by dense collagenous matrices that confer substantial mechanical strength and durability. These properties render them attractive candidates for scaffold development in diverse clinical contexts, including wound repair, urogenital reconstruction, vascular patches, orthopedic applications, and corneal repair [9,10,11,12,13,14,15]. However, the clinical use of native xenogeneic tissues is limited by the presence of immunogenic cellular elements that may elicit host immune responses and graft rejection [16]. Effective decellularization is therefore essential to eliminate cellular components while preserving ECM structure and function [17,18].

Chemical decellularization using detergents remains one of the most widely applied approaches. Surfactants such as sodium dodecyl sulfate (SDS) and Triton X-100 disrupt cellular membranes and solubilize intracellular components, facilitating efficient decellularization when appropriately optimized [6,19,20]. Triton X-100 is a delicate, non-ionic detergent that exerts minimal alterations in protein structure, while SDS is a potent, anionic detergent that denatures proteins with significant changes in the cell membrane’s integrity. Additionally, Triton X-100 tends to be less cytotoxic than SDS, making it more suitable for preserving the native structure and bioactivity of the ECM during tissue preparation [6]. On the other hand, SDS has stronger effects on the removal of lipids associated with the cell membrane and ECM, a step crucial for the successful production of scaffolds for tissue regeneration. Importantly, the success of decellularization protocols must be rigorously validated through macroscopic, histological, and ultrastructural analyses to ensure both cellular removal and ECM preservation [21]. These evaluations ensure that the decellularized scaffold is free of cellular components, structurally intact, and suitable for recellularization and implantation [22]. This study hypothesized that a sequential SDS and Triton X-100 decellularization protocol would achieve substantial cellular removal across tissues while preserving collagen integrity. The study aimed to evaluate the effectiveness of this protocol when applied to four ovine soft tissues: skin, tunica vaginalis, fascia lata, and pericardium. Comprehensive gross, histological, ultrastructural, and biochemical assessments were conducted to determine the suitability of the resulting decellularized tissues as scaffolds for tissue engineering and regenerative medicine applications.

## 2. Materials and Methods

### 2.1. Tissue Harvesting and Preparation

All ovine tissues used in this study were obtained from a licensed abattoir in Abu Khalifa, Ismailia Governorate, immediately following routine slaughter. No animals were sacrificed specifically for experimental purposes. Dorsal skin, tunica vaginalis, fascia lata, and pericardium were harvested from six healthy adult Ossimi rams. Tissue harvesting and preparation procedures were conducted following established protocols previously described for the respective tissue type [7,10,23,24]. Immediately after collection, tissues were immersed in ice-cold sterile phosphate-buffered saline (PBS; pH 7.4) supplemented with 1% penicillin–streptomycin (Gibco, Brooklyn, NY, USA) to minimize microbial contamination. Within 30 min of harvesting, samples were transported to the laboratory and processed for decellularization. Residual blood, adipose tissue, and non-structural connective tissues were carefully removed under sterile conditions. Samples were then sectioned into standardized 1 × 1 cm segments to ensure uniform decellularization and comparative analysis.

### 2.2. Decellularization Protocol

A sequential detergent-based decellularization protocol was applied as previously described [19], with minor modifications. All steps were performed at room temperature (22 ± 2 °C) under continuous agitation (50 rpm). Tissue samples were initially immersed in 0.5% (*w*/*v*) SDS (Sigma-Aldrich, Saint Louis, MO, USA) in deionized water for 48 h to disrupt lipid membranes and initiate cellular lysis. This was followed by incubation in 1% (*w*/*v*) SDS for an additional 24 h to ensure thorough solubilization of cellular and nuclear components. Subsequently, tissues were treated with 1% (*v*/*v*) Triton X-100 (Sigma-Aldrich, USA) for 1 h to facilitate removal of residual detergent and cellular debris.

After each detergent step, samples were washed three times in sterile PBS (pH 7.4) for 1 h per wash under constant agitation. Following decellularization, tissues were stored in sterile PBS containing 1% penicillin–streptomycin at 4 °C and used within 48 h for subsequent analyses. The average thickness of the used tissue segments was as follows: 1.7 mm (epidermis), 0.1 mm (tunica vaginalis), 1.2 mm (fascia lata), and 0.3 mm (pericardium).

### 2.3. Gross Morphological Evaluation

Decellularized and native tissue samples were independently examined by three investigators for changes in color, translucency, and surface texture.

### 2.4. Histological Analysis

Both native and decellularized tissues were fixed in 10% neutral-buffered formalin for 24 h, dehydrated through graded ethanol series, cleared in xylene, and embedded in paraffin. Sections (5 μm) were stained with hematoxylin and eosin (H&E) to evaluate tissue architecture and cellular removal. Mallory’s trichrome staining was performed to assess collagen distribution and ECM preservation [25].

### 2.5. Scanning Electron Microscopy (SEM)

To assess the ultrastructural organization and surface morphology of the native and decellularized tissues, SEM was performed as previously described [26,27]. Briefly, samples were fixed in 2.5% glutaraldehyde in 0.1 M phosphate buffer (pH 7.4) for 24 h at 4 °C. Fixed tissues were washed in PBS and dehydrated through a graded ethanol series (30% to 100%). Samples were then subjected to critical point drying using carbon dioxide and mounted on aluminum stubs. Tissues were sputter-coated with a thin layer of gold-palladium using a Quorum Q150R sputter coater (Quorum Technologies, East Sussex, UK) to ensure conductivity. Imaging was performed using a JSM-6510 LV scanning electron microscope (JEOL Ltd., Tokyo, Japan). Both surface and cross-sectional views were obtained at multiple magnifications to evaluate ECM morphology, porosity, and the absence of cellular components.

### 2.6. Residual DNA Quantification

Total DNA was extracted from decellularized and native tissue samples using the QIAamp DNA Mini Kit (Qiagen, Hilden, Germany) and quantified using Quant-iT PicoGreen dsDNA Assay Kit (Invitrogen, Carlsbad, CA, USA) per manufacturer instructions. Fluorescence was measured in a Synergy 2 microplate reader (BioTek Instruments Inc., Winooski, VT, USA) at an excitation wavelength of 480 nm and emission wavelength of 520 nm.

### 2.7. Statistical Analysis

Quantitative image analysis was performed to evaluate nuclear density and collagen fiber alignment in native and decellularized tissue samples. Nuclear density was determined by counting visible nuclei per unit area in H&E-stained sections using Fiji in ImageJ version1 (NIH, Bethesda, MD, USA), with mean values calculated for each sample. Collagen fiber alignment was assessed from high-resolution images, and the fiber alignment index was calculated using the Directionality plugin in ImageJ.

Tissues were obtained from six independent animals (*n* = six biological replicates). For each tissue type, one native and one decellularized sample were collected per animal, resulting in six independent samples per group. For histological analysis, three sections were examined per tissue sample. SEM was performed on samples from each animal to assess ultrastructural features. DNA quantification was conducted using one sample per tissue per animal. For all quantitative analyses, the statistical unit was the tissue sample derived from each individual animal.

Residual DNA content was quantified to assess decellularization efficiency. All quantitative data are presented as mean ± standard deviation (SD). Statistical comparisons between native and decellularized tissues were conducted using an unpaired Student’s *t*-test, with significance set at *p* < 0.05. All analyses were performed in GraphPad Prism version 9.0 (GraphPad Software, San Diego, CA, USA).

## 3. Results

### 3.1. Gross Morphological Alterations Following Decellularization

Gross examination revealed consistent and pronounced morphological changes across all four ovine tissues following decellularization. Compared with their native counterparts, decellularized samples exhibited increased translucency, reduced pigmentation, and smoother surface characteristics. These macroscopic changes were most evident in skin and pericardium, which transitioned from opaque, visibly cellular tissues to pale, semi-transparent matrices. In the skin, decellularization resulted in marked depigmentation and complete removal of hair shafts and follicular openings, yielding a uniform, flexible scaffold. Despite these alterations, all tissues retained their overall shape, thickness, and mechanical pliability, indicating preservation of the underlying extracellular matrix architecture while achieving effective cellular removal.

### 3.2. Histological Evaluation of Cellular Removal and ECM Preservation

**Figure 1:**
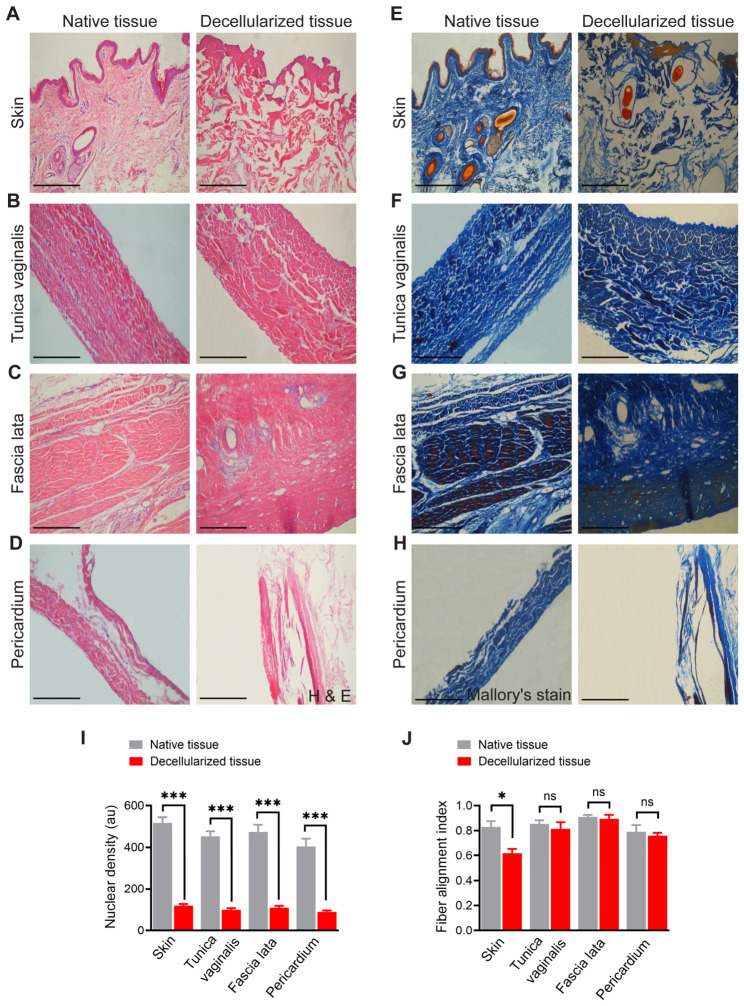
Histological evaluation of native and decellularized ovine tissues. (**A**–**D**) Hematoxylin and eosin (H&E) staining of native (**left**) and decellularized (**right**) tissues: (**A**) Skin: Native tissue shows a stratified squamous epithelium with hair follicles and dense connective tissue in the dermis. After decellularization, the epidermis is removed, and the dermal matrix remains with a marked loss of cellular components. (**B**) Tunica vaginalis: Native tissue demonstrates elongated fibroblast nuclei within aligned collagen bundles. Decellularized tissue retains the collagen fiber orientation but lacks nuclei, confirming effective removal of cellular material. (**C**) Fascia lata: Dense regular connective tissue in the native sample is characterized by parallel collagen fibers and visible fibroblast nuclei. Decellularized fascia lata preserves fiber alignment with no observable cellular content. (**D**) Pericardium: The native sample reveals a fibrous layer with visible nuclei, while the decellularized pericardium is acellular with a retained ECM structure. (**E**–**H**) Mallory’s trichrome staining of native (**left**) and decellularized (**right**) tissues: (**E**) Skin: Collagen fibers are stained blue in both native and decellularized tissues. (**F**) Tunica vaginalis: Blue-stained collagen is evident in both samples. Cellular details are visible in the native tissue, while decellularized tissue shows a uniform, acellular matrix. (**G**) Fascia lata: Dense blue collagen bundles with embedded nuclei in the native tissue are preserved structurally in the decellularized sample, but devoid of nuclear material. (**H**) Pericardium: Blue collagen fibers dominate the matrix in both samples. The decellularized pericardium appears acellular with well-maintained ECM integrity. (**I**) Quantitative analysis of the average nuclear density. (**J**) Quantitative analysis of the fiber alignment index. Data are presented as the mean ± SD. * *p* < 0.05, *** *p* < 0.001, and ns indicates non-significant. Scale bar = 200 µm.

Histological analyses using H&E and Mallory’s trichrome staining confirmed efficient decellularization and preservation of the collagen-rich ECM in all tissues (Figure 1). Native skin exhibited a well-organized stratified squamous epithelium with abundant adnexal structures, including hair follicles and sebaceous glands, embedded within a dense collagenous dermis. Following decellularization, the epidermal layer and all adnexal cellular components were completely removed. The dermal collagen network remained intact, although fibers appeared more loosely arranged, reflecting the absence of cellular tension (Figure 1A). Native tunica vaginalis displayed elongated fibroblast nuclei aligned parallel to dense collagen bundles, consistent with serosal connective tissue organization. In decellularized samples, nuclear material was absent, while collagen fiber orientation and lamellar structure were preserved (Figure 1B). Similarly, native fascia lata demonstrated densely packed, parallel collagen fibers with interspersed fibroblast nuclei, characteristic of dense regular connective tissue. Post-decellularization, the collagen alignment remained intact, with complete loss of cellular elements (Figure 1C). The native pericardium exhibited a multilayered fibrous architecture with scattered nuclei corresponding to mesothelial and stromal cells. Decellularized pericardial samples showed complete absence of nuclear staining, while maintaining the layered collagen framework (Figure 1D).

Mallory’s trichrome staining further confirmed these findings. In all tissues, blue staining highlighted collagen content. The native skin (Figure 1E) displayed both epithelial and dermal components with blue-stained collagen interwoven throughout. After decellularization, the epithelium and adnexal structures were absent, while the collagen matrix remained evident. Similarly, the tunica vaginalis (Figure 1F), fascia lata (Figure 1G), and pericardium (Figure 1H) retained collagen staining in their decellularized states, with an absence of cellular features, highlighting successful ECM preservation.

Quantitative histological analysis (Figure 1I) revealed a significant reduction in nuclear density in decellularized tissue compared to native tissue. In native samples, numerous intact nuclei were uniformly distributed throughout the tissue, indicating preserved cellular integrity. In contrast, decellularized tissues exhibited an absence or sparse presence of nuclear material, consistent with effective cellular removal. Statistical comparison confirmed that the nuclear density was markedly decreased in decellularized specimens (*p* < 0.001), validating the efficiency of the decellularization protocol in removing cellular components while preserving the overall matrix structure. In addition, fiber alignment analysis (Figure 1J) revealed tissue-specific differences. In skin samples, the fiber alignment index was significantly altered following decellularization, indicating disruption or reorganization of the extracellular matrix architecture (*p* < 0.05). However, no significant differences in fiber alignment were observed between native and decellularized forms of the tunica vaginalis, fascia lata, or pericardium, suggesting that decellularization preserved ECM orientation in these tissues.

### 3.3. Ultrastructural Assessment of Decellularized Tissues by Scanning Electron Microscopy

**Figure 2:**
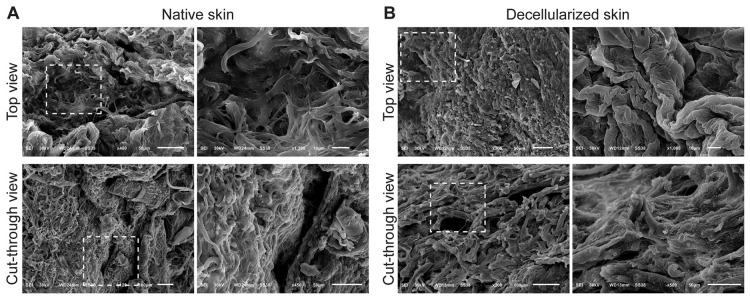
Scanning electron microscopy (SEM) analysis of native and decellularized sheep skin showing ultrastructural morphology from both surface and cross-sectional views. (**A**) Native skin: Top view: The surface appears dense and irregular, with prominent structural complexity and cell-like components embedded within the extracellular matrix. Cut-through view: A layered collagenous structure is observed with compact fibrous organization. Notably, a hair follicle structure is visible, indicating the presence of skin appendages and intact tissue architecture. (**B**) Decellularized skin: Top view: The surface is smoother and more organized compared to native tissue, with the absence of cells and a flattened topography consistent with ECM preservation. Cut-through view: The fibrous matrix remains intact with a porous and sponge-like appearance. The hair follicle is no longer visible, and the tissue appears fully devoid of cellular material. Dashed boxes in each panel highlight areas magnified in the adjacent images. Dashed boxes indicate regions of interest that are shown at higher magnification in the adjacent panels.

Ultrastructural assessment using SEM revealed major ultrastructural alterations in skin following decellularization. The top view of native skin exhibited a rough, irregular surface populated by cell-like protrusions consistent with keratinocytes and dermal fibroblasts. Cross-sectional views revealed stratified collagen layers and prominent hair follicle structures (Figure 2A). In contrast, decellularized skin displayed a smoother, flattened surface devoid of cellular features. The internal matrix exhibited a porous, sponge-like collagen architecture, indicating retention of the ECM framework following removal of epidermal and dermal cellular components, including hair follicle structures (Figure 2B).

**Figure 3:**
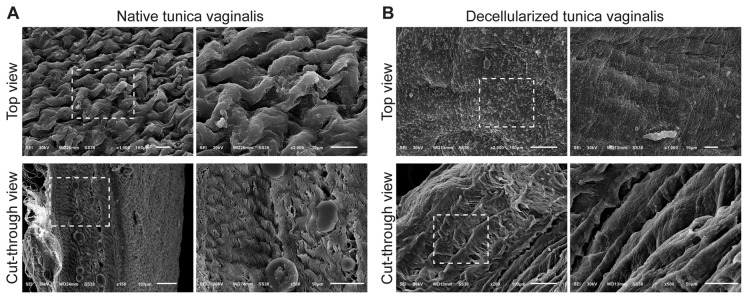
Scanning electron microscopy (SEM) images of native and decellularized sheep tunica vaginalis showing surface and internal ultrastructure. (**A**) Native tunica vaginalis: Top view: The surface exhibits a dense, undulating morphology with overlapping fibrillar layers and embedded cellular components, indicating an intact epithelial covering and compact ECM organization. Cut-through view: Cross-sectional images show a compact arrangement of collagen bundles with visible nuclei-like structures and blood vessel-like circular profiles, reflecting the organized native tissue architecture. (**B**) Decellularized tunica vaginalis: Top view: The surface becomes smoother and more aligned with parallel ECM fibers and the absence of cells, indicating successful removal of epithelial and stromal cells. Cut-through view: Internal structure remains intact with preserved ECM fibers that appear more porous and loosely organized compared to the native tissue, confirming the structural integrity of the scaffold post-decellularization. Dashed boxes indicate regions magnified in adjacent high-resolution views. Dashed boxes indicate regions of interest that are shown at higher magnification in the adjacent panels.

The surface of native tunica vaginalis (Figure 3A) showed a dense, undulating surface with overlapping fibrillar layers and visible cellular elements. Cross-sectional SEM images showed compact collagen bundles with occasional circular structures, possibly representing vascular lumens or glandular profiles. Elongated nuclei-like structures were visible, indicating viable fibroblasts and a well-organized stromal layer. After decellularization, the surface became smoother and more uniformly aligned, with no detectable cellular remnants. Internally, collagen bundles remained well organized, albeit with increased porosity (Figure 3B), suggesting successful clearance of vascular and stromal cells while preserving ECM alignment and overall structural integrity.

**Figure 4:**
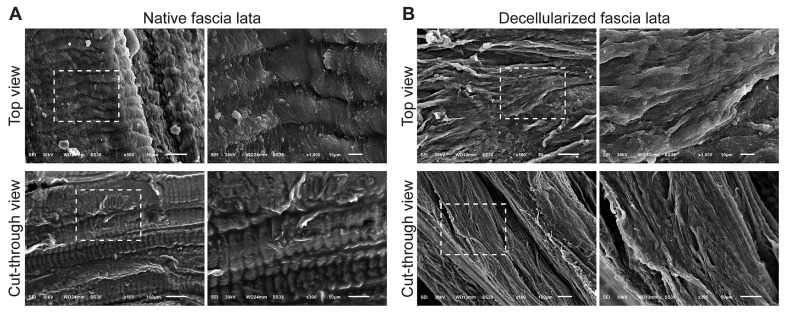
Scanning electron microscopy (SEM) images showing surface and internal ultrastructure of native and decellularized sheep fascia lata. (**A**) Native fascia lata: Top view: The surface of the native fascia lata shows highly organized, parallel collagen fibers with distinct striations and tightly packed fibrous bundles. Cellular residues and microstructural irregularities are also visible, indicating the presence of native cellular components. Cut-through view: The cross-sectional architecture reveals compact, layered collagen lamellae arranged in parallel alignment, with clearly distinguishable dense ECM and occasional embedded cells. (**B**) Decellularized fascia lata: Top view: After decellularization, the surface maintains the overall alignment of collagen fibers, though the tissue appears smoother and more fibrillar, with the absence of visible cellular components. Cut-through view: The ECM structure remains well-preserved, with more prominent and distinct collagen fiber layers and increased porosity, indicating effective removal of cells while maintaining the native architecture. Dashed boxes indicate regions of interest that are shown at higher magnification in the adjacent panels.

The native fascia lata demonstrated highly aligned, parallel collagen fibers forming dense lamellae with occasional cellular remnants (Figure 4A). Decellularized fascia lata retained its characteristic parallel collagen organization while exhibiting smoother fibrillar surfaces and increased interfibrillar spacing, indicative of enhanced porosity without disruption of matrix alignment (Figure 4B). The cross-sectional structure revealed prominent, well-preserved lamellae and an increase in interfibrillar spacing, suggesting enhanced porosity post-decellularization without compromising matrix architecture.

**Figure 5:**
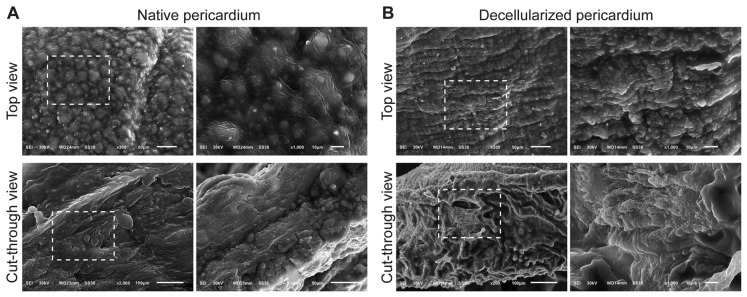
Scanning electron microscopy (SEM) images of native and decellularized sheep pericardium showing surface morphology and internal structural integrity. (**A**) Native pericardium: Top view: The surface exhibits a dense and uneven texture with numerous dome-shaped protuberances, corresponding to mesothelial cells that form the outer epithelial lining of the pericardium. Beneath this layer, the extracellular matrix (ECM) appears compact and well-organized. Cut-through view: The cross-sectional architecture reveals a multilayered, fibrous matrix with tightly packed collagen bundles and cellular inclusions, indicating intact native tissue. (**B**) Decellularized pericardium: Top view: The mesothelial layer is absent, and the surface appears smoother with exposed collagen fibers and an aligned ECM pattern, indicating effective removal of cellular material. Cut-through view: The fibrous structure of the ECM remains well-preserved, with a porous and sponge-like appearance, confirming that the decellularization process maintained the integrity of the collagen framework. Dashed rectangles indicate regions magnified in adjacent high-resolution views. Dashed boxes indicate regions of interest that are shown at higher magnification in the adjacent panels.

Native pericardium presented a dense surface with dome-shaped protrusions corresponding to mesothelial cells. These features were completely absent in decellularized samples, which instead displayed exposed, aligned collagen fibers. Cross-sectional views confirmed preservation of the multilayered fibrous ECM with increased porosity and complete absence of nuclear or cytoplasmic structure (Figure 5A,B).

### 3.4. Assessment of Residual DNA Content

**Figure 6:**
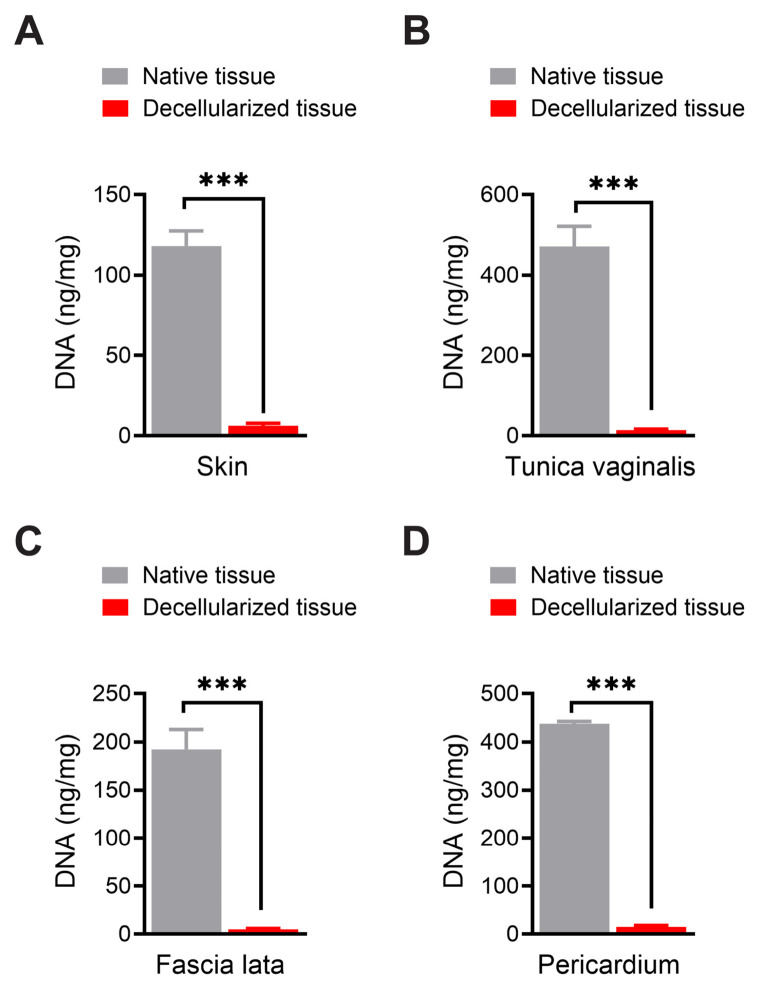
Quantitative comparison of DNA content of native and decellularized tissues of sheep. (**A**) Skin. (**B**) Tunica vaginalis. (**C**) Fascia lata. (**D**) Pericardium. *** *p* < 0.001.

Quantitative DNA analysis demonstrated elimination of nucleic acid content in all decellularized tissues (Figure 6). Compared with native tissues, residual DNA was reduced by approximately 94.1% in skin, 95.0% in tunica vaginalis, 94.5% in fascia lata, and 93.5% in pericardium. These reductions were statistically significant (*p* < 0.001), confirming effective removal of potentially immunogenic cellular material.

## 4. Discussion

The present study demonstrates that a sequential detergent-based protocol using SDS and Triton X-100 effectively decellularizes multiple ovine soft tissues while preserving their extracellular matrix architecture. Through combined gross, histological, ultrastructural, and biochemical analyses, we show that skin, tunica vaginalis, fascia lata, and pericardium can be reliably converted into acellular scaffolds suitable for tissue engineering applications.

Gross morphological changes, including increased translucency and loss of pigmentation, are widely recognized indicators of successful decellularization and were consistently observed across all tissues. These macroscopic features reflect the removal of cellular and vascular components while maintaining the collagenous framework, in agreement with previous reports on decellularized biological scaffolds [28,29].

Consistent with gross observations, histological staining further confirmed the gross observations. In native tissues, dense nuclei and cytoplasmic structures were visible, particularly in skin (with hair follicles), fascia lata (dense fibroblasts), and tunica vaginalis (elongated fibroblasts). After decellularization, these features were absent, with a clear loss of nuclei and cytoplasmic material, confirming the efficacy of SDS and Triton X-100 in cellular removal [10,16,22]. Notably, Mallory’s trichrome staining revealed intact and well-preserved collagen networks across all decellularized tissues, with strong blue staining in both superficial and deep layers. This retention of the collagen framework is essential for the mechanical strength and biological signaling potential of ECM scaffolds, and it reinforces the utility of the chosen protocol, which effectively balances decellularization with structural preservation [30,31].

Ultrastructural analysis via SEM supported these findings by revealing surface irregularities, cell-associated structures (e.g., domed mesothelial cells in pericardium, hair follicles in skin), and dense fibrous ECM in the native tissues. However, the tissue surfaces appeared smoother, more fibrillar, and devoid of cells after decellularization. Cut-through views showed well-organized, porous collagen layers, indicating minimal disruption to the ECM’s three-dimensional architecture. In the decellularized pericardium, the absence of dome-shaped mesothelial cells and retention of collagen bundles reflect effective decellularization with mesothelial layer removal. Similarly, in fascia lata and tunica vaginalis, the collagen fibers retained their alignment, suggesting these tissues may have the potential to support uniaxial loading; however, this requires confirmation through biomechanical testing. These results are consistent with prior studies emphasizing the importance of ECM microarchitecture for cellular infiltration, angiogenesis, and tissue integration after implantation [9,16,30].

Among the four tissues, fascia lata exhibited the most robust and densely organized collagenous structure, suggesting potential suitability for load-bearing applications, pending further validation such as ligament or tendon reconstruction [32]. The pericardium, though thinner, demonstrated a porous yet well-organized matrix, making it a potential scaffold for cardiac, corneal, or serosal tissue repair [33,34,35]. Skin, with its layered collagen and previous presence of adnexal structures like hair follicles, provides a promising platform for dermal substitutes [36]. Tunica vaginalis, due to its pliability and bi-layered collagen arrangement, may serve as a potential candidate for reconstructive esophageal, urological, or abdominal wall applications [9,10,37,38]. Furthermore, the efficient removal of immunogenic components (e.g., nuclear material and cell membranes) suggests reduced cellular content, which may contribute to lower immunogenic potential; however, further studies are required to assess antigenicity and host immune response [39,40].

Limitations of the present study include the absence of biomechanical testing, comprehensive biochemical characterization of ECM components, and in vivo implantation studies. In the present study, ECM preservation was evaluated using histological staining and SEM, which demonstrated effective cellular removal and maintenance of overall tissue architecture and collagen organization. However, these approaches primarily provide structural information and do not fully characterize the biochemical composition of the ECM. Specifically, quantitative assessment of key matrix components such as glycosaminoglycans, elastin, fibronectin, laminin, and basement membrane-associated proteins was not performed. Given that detergent-based decellularization, particularly with SDS, may alter or extract bioactive ECM constituents, this represents an important limitation of the current study.

Future investigations should address these aspects, along with recellularization efficiency and host immune responses, to further validate the clinical utility of these scaffolds. Furthermore, quantitative assessment of residual detergents should be included in future studies to ensure biosafety.

## 5. Conclusions

These findings demonstrate effective decellularization and preservation of extracellular matrix structure in multiple ovine tissues. While the resulting scaffolds show promising structural characteristics, further biomechanical, biochemical, and in vivo studies are required to confirm their suitability for tissue engineering and regenerative medicine applications.

## Data Availability

Data is contained within the article.

## Abbreviations

The following abbreviations are used in this manuscript:

dECM	Decellularized extracellular matrix
ECM	Extracellular matrix
SEM	Scanning electron microscopy
SDS	Sodium dodecyl sulfate
H&E	Hematoxylin and eosin
PBS	Phosphate-buffered saline
